# Beyond the bottle: exposing the identity-rate gap in microzooplankton ecology

**DOI:** 10.1093/plankt/fbag070

**Published:** 2026-09-01

**Authors:** Albert Calbet

**Affiliations:** Institut de Ciències del Mar (ICM-CSIC), Department of Marine Biology and Oceanography, Passeig Marítim de la Barcelonta 37-49, 08003, Barcelona, Spain

**Keywords:** microzooplankton, dilution technique, grazing, preservation, trait-based ecology

## Abstract

Microzooplankton ecology was transformed by the dilution method, which made grazing measurable in natural plankton communities and established protistan grazers as major consumers of marine primary production. Four decades later, the field faces a different challenge. Automated imaging, metabarcoding, meta-omics and machine-learning tools increasingly resolve plankton identity, diversity and spatial structure, but grazer-resolved rates of ingestion, growth, prey selectivity, mortality and carbon transfer remain much harder to measure. This creates an identity-rate gap: community structure is becoming easier to describe than ecological function. The gap should not be reduced to a contrast between one classical method and modern identity tools; it reflects a broader difficulty of pairing organisms, prey, rates and environmental context within the same inference framework. Dilution experiments and related rate approaches remain indispensable, but their interpretation depends on assumptions about prey growth, grazer responses, nutrient limitation, mixoplankton behavior and bottle effects. Preserved samples also remain central to biomass estimates, although fixation and storage distort protists in taxon-specific ways. I argue that the next phase of microzooplankton ecology should not abandon classical incubations, but make them identity-aware, preservation-aware and model-facing through tiered datasets that separate bulk prey mortality, prey-resolved rates, targeted grazer-prey links and model-facing trait constraints.

## INTRODUCTION: FROM A RATE REVOLUTION TO A RATE-IDENTITY IMBALANCE

Microzooplankton ecology has always been shaped by measurement problems. Phytoplankton production can be estimated, chlorophyll can be mapped, and plankton cells can be counted, but the disappearance of newly produced organic matter into protistan consumers is much harder to observe directly. Before experimental rate methods became routine, microzooplankton grazing was easy to underestimate because it left few obvious traces. The development of the dilution method by Landry and Hassett (1982) changed that situation. By experimentally reducing encounter rates between prey and grazers while estimating phytoplankton growth, the method made it possible to infer community-level grazing pressure in natural assemblages. It turned microzooplankton grazing from a plausible process into a measurable rate.

This achievement was conceptual as well as technical. The dilution method appeared just before the microbial loop concept of Azam *et al.* (1983), and the two developments together helped reframe bacteria and protists as active components of pelagic food webs rather than background microbial biomass. Dilution experiments and subsequent syntheses then showed that microzooplankton are not peripheral consumers but often remove a substantial fraction of daily primary production (Calbet and Landry, 2004; Schmoker *et al.,* 2013). In many marine systems, a large part of the carbon fixed by phytoplankton is processed through protistan grazers before it can be exported, transferred to metazoans or recycled. Therefore, this method helped reposition microzooplankton as regulators of phytoplankton standing stocks, nutrient regeneration and carbon routing.

That success now creates a new imbalance. The operational category microzooplankton includes aloricate and loricate ciliates, heterotrophic and mixotrophic dinoflagellates, radiolarians, small metazoan stages, flagellates and other protists with distinct feeding modes, prey spectra, growth efficiencies and preservation sensitivities. A bulk grazing coefficient, even when robust, compresses that diversity into a single loss term. Such compression is useful for many comparative and modelling purposes, but it becomes increasingly inadequate as the field gains a sharper view of plankton identity and structure.

The observational side of plankton ecology has changed dramatically. FlowCAM, Imaging FlowCytobot and other imaging systems can generate large numbers of plankton images and classify them semi-automatically or automatically (Sieracki *et al.,* 1998; Olson and Sosik, 2007; Sosik and Olson, 2007; Álvarez *et al.,* 2014). Global and regional metabarcoding surveys have revealed extensive eukaryotic plankton diversity, including cryptic lineages and biogeographic structure that were poorly resolved by conventional microscopy (de Vargas *et al.,* 2015; Massana *et al.,* 2015; Burki *et al.,* 2021). *In situ* imaging has also revealed large and fragile protists that are difficult to sample by bottles or nets (Biard *et al.,* 2016). These tools have expanded the identity axis of the field, while recent deep-learning approaches further increase the speed and scale of image-based plankton analysis (Bachimanchi *et al.,* 2024).

Functional rates have not expanded at the same pace. We can often describe community composition, image classes or sequence variants at high resolution, but we still struggle to assign ingestion, prey selectivity, growth efficiency and carbon transfer to particular grazers or functional groups. This Perspective calls that mismatch the identity-rate gap (Table I). The gap is not a claim that papers citing the original dilution method exhaust rate-based knowledge. Rate-relevant work also includes non-dilution and hybrid approaches: disappearance or uptake of fluorescent prey, live fluorescently labelled algae (LFLA), food-vacuole or prey-content observations, stable-isotope or other tracer incubations, flow-cytometric prey-loss measurements, behavioral imaging, sorted-grazer prey DNA/RNA, pigment-resolved assays, molecularly resolved dilution variants and model-based inference (Sherr and Sherr, 1987; Worden and Binder, 2003; Martínez *et al.,* 2014; Landry *et al.,* 2024a; Beatty *et al.,* 2025; Gu *et al.,* 2026). The gap is the situation in which identity becomes rich while rate information remains bulk, indirect, preservation-sensitive or poorly linked to traits. Closing it requires more than adding molecular samples to incubations; it requires a measurement framework that connects identity, preservation, prey tracking, experimental rates and model parameters. Recent field applications continue to demonstrate the value of dilution-based rate measurements under contrasting regional and perturbation contexts (Wen *et al.,* 2023; Landry *et al.,* 2024b), but they also show how rarely such rates are paired with grazer-level identity.

**Table I:** Key literature lines and their implications for the identity-rate gap

Literature line	Representative references	What it established	What remains unresolved
Dilution experiments and rate extensions	Landry and Hassett, 1982; Landry *et al.,* 1995; Worden and Binder, 2003; Calbet and Landry, 2004; Schmoker *et al.,* 2013; Morison and Menden-Deuer, 2017; Wen *et al.,* 2023; Landry *et al.,* 2024a, 2024b; Beatty *et al.,* 2025; Gu *et al.,* 2026	Established microzooplankton grazing as a major loss pathway for primary production and showed that dilution logic can be extended to prey groups, flow-cytometric populations and molecularly resolved subpopulations.	Often bulk or prey-proxy based; even prey-resolved mortality does not by itself identify the grazer or solve the full interaction matrix.
Dilution critiques and refinements	Dolan *et al.,* 2000; Dolan and McKeon, 2005; Landry and Calbet, 2005; Agis *et al.,* 2007; Taniguchi *et al.,* 2012; Calbet and Saiz, 2013, 2018; Beckett and Weitz, 2017; Li *et al.,* 2017; Beatty *et al.,* 2025	Clarified assumptions about nutrient effects, nonlinear responses, trophic cascades, sampling effort, method accuracy and growth-rate inference.	Shows that rates are conditional outcomes of experimental perturbations and design choices, not automatically transferable constants.
Non-dilution and single-cell rate proxies	Sherr and Sherr, 1987; Rublee and Gallegos, 1989; Hammer *et al.,* 2001; Jezbera *et al.,* 2005; Zubkov and Tarran, 2008; Martínez *et al.,* 2014; Brown *et al.,* 2020; Bachimanchi *et al.,* 2024; Beatty *et al.,* 2025w	Linked activity to prey or grazer identity through fluorescent prey uptake or disappearance, tracer particles, flow-cytometric prey loss, food-vacuole/prey-content evidence, single-cell molecular evidence, behavioral imaging and tracers.	Prey-proxy specific and calibration dependent; some snapshots lack full community denominators, time denominators or carbon-flux conversion.
Imaging and automated classification	Sieracki *et al.,* 1998; Olson and Sosik, 2007; Sosik and Olson, 2007; Álvarez *et al.,* 2014; Orenstein *et al.,* 2015; Biard *et al.,* 2016; Bachimanchi *et al.,* 2024	Greatly expanded structural and temporal observation of plankton.	Images provide identity, size and morphology but not ingestion, growth or carbon-transfer rates without calibration or prey tracking.
Molecular diversity and metabarcoding	de Vargas *et al.,* 2015; Massana *et al.,* 2015; Santoferrara *et al.,* 2020; Burki *et al.,* 2021; Weydmann-Zwolicka *et al.,* 2024; Landry *et al.,* 2024a; Gu *et al.,* 2026	Revealed hidden diversity, cryptic taxa, biogeographic structure and, in modified designs, potential subpopulation-level rate information.	Sequence data do not directly provide cell abundance, biomass, feeding rates, behavior or carbon flux unless linked to abundance calibration, experiments or prey tracking.
Mixoplankton and trophic mode	Stoecker, 1998; Flynn and Mitra, 2009; Flynn *et al.,* 2013, 2019; Mitra *et al.,* 2016; Stoecker *et al.,* 2017; Millette *et al.,* 2023; Mitra *et al.,* 2023	Reframed many protists as flexible phototrophic-phagotrophic organisms.	Complicates phytoplankton-versus-zooplankton assumptions in experiments and models; different mixoplankton functional types may require different measurements.
Preservation artifacts	Bloem *et al.,* 1986; Vaulot *et al.,* 1989; Choi and Stoecker, 1989; Sherr and Sherr, 1993; Leakey *et al.,* 1994; Stoecker *et al.,* 1994; Pfister *et al.,* 1999; Menden-Deuer *et al.,* 2001; Chaput and Carrias, 2002; Karayanni *et al.,* 2004; Modigh and Castaldo, 2005; Zarauz and Irigoien, 2008; Sano *et al.,* 2020; Nogueira *et al.,* 2023; Calbet, 2026	Showed that fixation alters abundance, volume, biomass and taxonomic detectability.	State variables used to interpret rates are method-dependent and should carry preservation metadata and uncertainty.
Models and carbon-cycle uncertainty	Follows *et al.,* 2007; Ward *et al.,* 2012, 2014; Rohr *et al.,* 2023; Meyjes *et al.,* 2024	Demonstrated the need for trait-based and better constrained grazing formulations.	Models cannot use every taxonomic detail; empirical rates must become model-facing, categorized and uncertainty-aware.
Feeding mechanisms and carbon routing	Kiørboe, 2011; Mitra *et al.,* 2014; Ward and Follows, 2016; Steinberg and Landry, 2017	Showed that consumer feeding mode, mixotrophy and zooplankton processing pathways shape trophic transfer, recycling and export.	Microzooplankton rate data are rarely linked to pathway-specific carbon fate or grazer-prey mechanisms.

The issue has consequences beyond microbial ecology, but the model connection must be stated realistically. Marine biogeochemical and Earth system models represent grazing as a central pathway controlling phytoplankton biomass, export, remineralization and carbon sequestration, yet most models cannot accommodate every taxon, prey choice or grazer behavior observed in the field. Recent work has shown that zooplankton grazing terms are a major source of uncertainty in modelled marine carbon cycling (Rohr *et al.,* 2023), and that spatial variability in grazing parameters can strongly affect carbon export estimates (Meyjes *et al.,* 2024). These studies do not focus exclusively on microzooplankton, but they expose the broader modelling problem: grazing is both ecologically central and empirically underconstrained. The practical contribution of microzooplankton ecology is therefore not a perfectly resolved interaction matrix, but better constrained prey-size-specific mortality, functional-response parameters, trait ranges and uncertainty estimates that models can actually use.

## WHAT DILUTION EXPERIMENTS ESTABLISHED, AND WHAT THEY STILL LEAVE UNRESOLVED

A constructive critique must begin with what dilution experiments established. The method was designed to estimate phytoplankton growth and grazing mortality in mixed natural communities, not to identify every interaction within them. Its power lies in integration. A dilution experiment asks how the apparent growth of a prey community changes as encounter rates with grazers are reduced. Under the classical assumptions, the intercept provides phytoplankton growth and the slope provides grazing mortality. That simple regression converted complex microbial interactions into a tractable field measurement and generated the comparative evidence base on which much of modern microzooplankton ecology rests.

The broad conclusion from that evidence remains robust: microzooplankton frequently exert strong top-down control on phytoplankton. Calbet and Landry (2004) synthesized global evidence and argued that microzooplankton grazing is a dominant pathway for primary production through marine food webs. Schmoker *et al.* (2013) later compiled a much larger dataset and showed that grazing impacts are large but highly variable across environments. Some of this variability is ecological signal, reflecting differences in productivity, prey size, grazer composition, nutrient regime, temperature, bloom stage and food-web structure. Some of it also reflects methodological heterogeneity, because historical studies differed in dilution design, nutrient amendment, incubation light, bottle handling, endpoint choice and correction for incubation artifacts such as photoacclimation. For syntheses and model parameterization, both sources of variability matter; not every difference among grazing estimates can be interpreted as an environmental effect.

The limitations of the dilution method arise from the same integration that makes it useful. A standard chlorophyll-based dilution experiment measures the net response of a prey proxy to manipulated grazer-prey encounter rates. It usually does not reveal which grazers were responsible, which prey were preferentially consumed, whether feeding was saturated for the different grazers, whether prey growth differed among dilution levels or whether predators of microzooplankton altered the result. These are not minor details. They determine whether a grazing estimate should be interpreted as a community-average loss rate, a prey-specific mortality rate, a grazer-specific feeding rate or a conditional outcome of a manipulated food web.

This distinction between rate currencies is central. A dilution-derived slope estimated from chlorophyll a is not the same object as a prey-group mortality rate, a grazer-specific ingestion rate or a biomass-normalized carbon-transfer coefficient. All may be useful, but they answer different questions. Problems arise when bulk chlorophyll loss is later compared with prey-resolved or grazer-linked estimates as if all represented the same rate currency. The next generation of experiments should therefore report the inferential level of each rate: community-level mortality, prey-specific loss, grazer-resolved ingestion, functional-group activity or model-fitted parameter.

Several lines of methodological work have clarified these issues. Landry *et al.* (1995) refined the technique and emphasized design choices such as nutrient additions, dilution levels and taxon-specific measurements, while taxon-focused applications to Prochlorococcus and Synechococcus showed how dilution designs can be adapted beyond bulk chlorophyll (Worden and Binder, 2003). Dolan *et al.* (2000), Dolan and McKeon (2005) and Agis *et al.* (2007) raised concerns about dilution effects, bottle-driven microbial community dynamics and the possibility that organic carbon consumption could be overestimated. Landry and Calbet (2005) responded by defending the method while emphasizing the need for reality checks and appropriate interpretation. Later analyses showed that nonlinear responses can arise when grazing is saturated, when trophic cascades occur within incubation bottles or when prey growth differs among treatments (Taniguchi *et al.,* 2012; Calbet and Saiz, 2013; Li *et al.,* 2017). Beckett and Weitz (2017) further showed that niche competition among phytoplankton can complicate inference when grazing is not the only process shaping apparent growth, and sampling-design analyses have emphasized that rate precision depends on effort and temporal resolution (Morison and Menden-Deuer, 2017). More recent studies have extended dilution logic by combining it with 16S rRNA sequence information, flow-cytometric populations or direct method comparisons, and method-comparison work has also made clear why non-dilution prey-disappearance or tracer approaches must be treated as complementary rather than interchangeable rate currencies (Landry *et al.,* 2024a; Beatty *et al.,* 2025; Gu *et al.,* 2026). These debates and extensions did not invalidate dilution experiments. They revealed that their assumptions are ecological hypotheses, not automatic conditions.

Nutrient addition illustrates the point. Nutrients are often added to avoid differential nutrient limitation along dilution series, because the assumption of equal phytoplankton growth across treatments is central to the classical interpretation. Yet nutrient enrichment can itself alter growth, prey physiology, food quality, mixotrophic behavior and grazer-prey coupling. Calbet and Saiz (2018) argued that insufficient nutrient addition can severely inflate grazing estimates, while excessive or poorly justified enrichment may create unrealistic incubation conditions. The solution is not to avoid nutrients reflexively or to add them blindly, but to report and justify the nutrient design and to evaluate whether it changes the process being measured.

Trophic cascades provide a second example. Dilution bottles do not contain only phytoplankton and microzooplankton. They may also contain predators of grazers, parasites, viruses and competitors. Changing the fraction of whole seawater changes not only grazer-prey encounter rates but also the abundance of organisms that modify grazer activity. Calbet and Saiz (2013) showed that trophic cascades can generate artificial saturation curves, V-shaped relationships or even positive slopes. Such patterns are sometimes treated as failed experiments, but they can also contain information about food-web structure. The field should therefore move from a binary view of valid versus invalid dilution experiments toward a diagnostic view: what ecological processes produce the observed response curve, and what rate can defensibly be inferred from it?

Mixoplankton complicate the classical framework even further. If an organism can photosynthesize and feed, its response to dilution may not fit a simple prey-versus-grazer partition. Dilution changes prey availability, light exposure per cell, nutrient competition, bacterial abundance and grazer-prey ratios, all of which may alter the balance between phototrophy and phagotrophy. Ferreira *et al.* (2021) showed that mixoplankton can interfere with dilution estimates, while the wider mixoplankton literature argues that plankton functional categories based on a strict phytoplankton-zooplankton dichotomy are inadequate (Flynn and Mitra, 2009; Flynn *et al.,* 2013, 2019; Stoecker *et al.,* 2017; Millette *et al.,* 2023). For microzooplankton ecology, this means that a grazing experiment can simultaneously manipulate consumers that are also primary producers or nutrient scavengers.

The most useful future role of dilution experiments is therefore not to treat them as direct, unaltered snapshots of natural grazing, but as controlled experimental manipulations. Dilution changes the natural assemblage by reducing grazer-prey encounter rates and, in some cases, by modifying nutrient conditions, predator–prey ratios, trophic cascades or bottle environments. These changes do not make the method invalid, but they define what the method can and cannot tell us. A dilution experiment is most powerful when its assumptions are explicit, its possible artifacts are evaluated, and the rate being inferred is clearly defined: bulk community grazing, prey-specific mortality, grazer-specific ingestion or a parameter intended for models. A chlorophyll-only dilution experiment can still provide a valuable bulk estimate. A stronger experiment pairs chlorophyll with flow cytometry, microscopy, imaging or molecular information. A still stronger design tracks prey groups, grazer composition and post-incubation community changes. The future is not to abandon dilution, but to increase its inferential resolution while making the level of inference explicit.

## A REALISTIC TIERED DESIGN FOR IDENTITY-AWARE DILUTION EXPERIMENTS

**Fig. 1:**
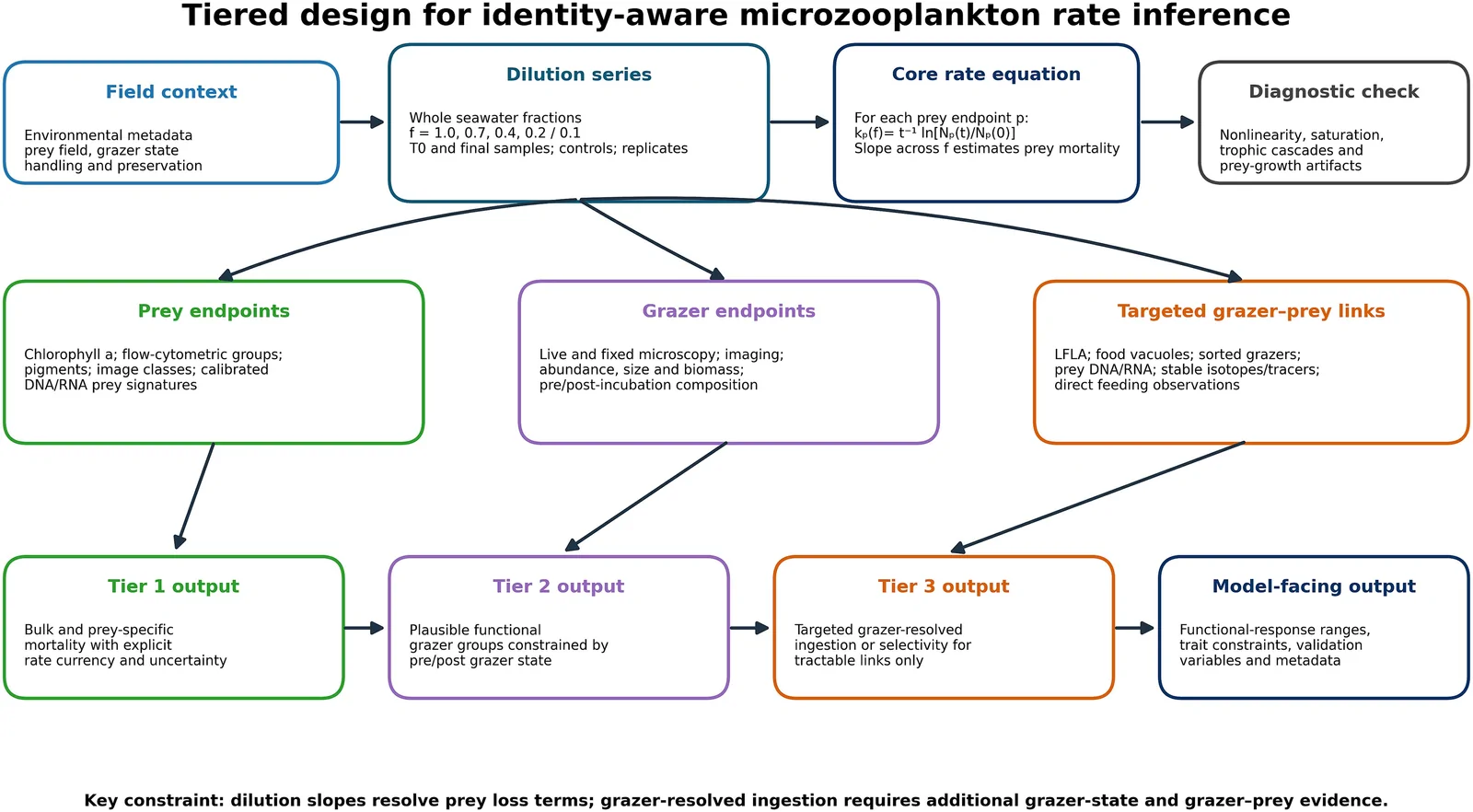
Tiered workflow for identity-aware rate inference. The figure separates the minimum dilution-based calculation of prey-specific mortality from the additional observations required for grazer-state inference, targeted grazer-prey links and model-facing parameter constraints. It emphasizes that not every study must apply every tier; the measurement package should match the rate currency being claimed. Alt-text: Flowchart showing a field sample entering a dilution series, followed by prey measurements, grazer measurements and targeted prey-tracking observations that support increasingly resolved levels of inference from bulk mortality to model-facing trait constraints.

The design proposed here is deliberately tiered; it is not a request that every study estimate every pairwise interaction in a microbial food web. A workable field experiment begins with the classical dilution series, for example several fractions of whole seawater spanning undiluted to strongly diluted treatments, with replicate bottles, time-zero and final samples, stated nutrient additions or unenriched controls, and environmental metadata for the sampled water parcel. The first design decision is the rate currency: bulk chlorophyll mortality, prey-group mortality, grazer-state change, targeted ingestion or a model parameter. That decision determines which extra measurements are required. In that sense, Fig. 1 is a decision tree rather than a demand for maximal sampling. This tiered logic follows refined dilution designs and sampling-effort analyses rather than treating extra measurements as mandatory in every bottle (Landry and Hassett, 1982; Landry *et al.,* 1995; Morison and Menden-Deuer, 2017; Beatty *et al.,* 2025).

For each measured prey class p, the defensible first-level calculation remains transparent: k_p(f)_ = t^{−1}^ ln[N_p(t)_/N_p(0_)], where f is the fraction of whole seawater and N_p is the abundance, biomass, pigment or molecularly calibrated abundance proxy for prey class p. Under the linear, unsaturated version of the dilution model, the intercept estimates prey growth and the slope estimates grazer-mediated mortality for that prey class. This produces a vector of prey-specific mortality rates, not a full grazer-by-prey interaction matrix. The same prey-class formulation underlies taxon- or population-focused dilution applications to picophytoplankton and sequence-resolved microbial subpopulations (Worden and Binder, 2003; Landry *et al.,* 2024a; Gu *et al.,* 2026), while nonlinear designs require additional diagnostic treatment (Li *et al.,* 2017).

Grazer-side inference requires additional equations and independent observations. Pre/post incubation counts, biomass and image or sequence data for grazer functional groups can estimate net grazer change and constrain which consumers were present, increasing or disappearing. They do not by themselves reveal which grazer ate which prey. Targeted grazer-prey links require extra observables, such as LFLA, food-vacuole inspection, prey DNA or RNA in sorted grazers, stable-isotope or other tracers, or direct behavioral observations. For pallium- or peduncle-feeding dinoflagellates, for example, a dilution slope can identify loss of large target prey, but ingestion by that feeding mode is only supported when paired with direct microscopic, tracer or prey-remnant evidence. These links have precedent in fluorescent bacteria or algae, food-vacuole/FISH approaches, tracer-particle methods and single-cell molecular prey-content analyses (Sherr and Sherr, 1987; Rublee and Gallegos, 1989; Hammer *et al.,* 2001; Jezbera *et al.,* 2005; Martínez *et al.,* 2014; Brown *et al.,* 2020).

A practical program therefore has three tiers. Tier 1 is a defensible bulk or prey-resolved mortality experiment with full design and uncertainty reporting. Tier 2 adds grazer composition, live/fixed biomass estimates and post-incubation diagnostics to identify plausible consumer groups and bottle effects. Tier 3 targets a small number of dominant or mechanistically important grazer-prey links with tracers, sorting, imaging or molecular prey tracking. The tiers are cumulative only when the biological question justifies the added effort. The point is not to solve every unknown from one bottle, but to avoid confusing what has been measured with what has merely been inferred (Fig. 1). Similar combinations of prey-resolved dilution, tracer or molecular information have already been used in partial form, which is why the proposed tiers formalize existing practice rather than present a wholly new method (Worden and Binder, 2003; Martínez *et al.,* 2014; Landry *et al.,* 2024a; Beatty *et al.,* 2025; Gu *et al.,* 2026).

Complementary non-dilution approaches should be treated as rate anchors in their own right, not as minor appendices to dilution experiments. Fluorescent prey disappearance, labelled-prey uptake, food-vacuole or prey-remnant observations, sorted-grazer prey DNA/RNA, stable-isotope or other tracer additions, flow-cytometric prey-loss measurements and behavioral imaging can provide stronger prey or grazer specificity than a bulk chlorophyll slope. They also carry their own limitations: surrogate prey may be selected differently from natural prey, uptake snapshots may lack a community-level denominator, prey DNA can reflect recent ingestion or contamination, and conversion to carbon flux requires calibration. The practical rule is therefore to report each method’s rate currency explicitly and to use cross-method agreement as evidence, not to force all methods into a single apparent grazing coefficient. This framing is consistent with method-comparison work and with studies using labelled prey, food-vacuole/prey-content evidence, single-cell genomics and individual-level imaging to connect identity with activity (Sherr and Sherr, 1987; Rublee and Gallegos, 1989; Jezbera *et al.,* 2005; Martínez *et al.,* 2014; Brown *et al.,* 2020; Bachimanchi *et al.,* 2024; Beatty *et al.,* 2025).

## FROM BULK GRAZING COEFFICIENTS TO GRAZER-RESOLVED MECHANISMS

The identity-rate gap becomes most visible when the question shifts from how much phytoplankton mortality occurred to who caused it and through which pathway. Microzooplankton are not a homogeneous grazing compartment, a point consistent with earlier plankton food-web and microzooplankton syntheses that treated protists as functionally diverse mediators of carbon flow rather than a single grazing box (Fenchel, 1988; Calbet, 2008). Ciliates, heterotrophic dinoflagellates and mixoplankton can have different prey-size spectra, feeding mechanisms, encounter strategies, growth efficiencies and vulnerability to predators. They also route carbon differently. Some feeding interactions recycle carbon rapidly through the microbial loop; others transfer larger particles to metazoans; others fragment cells, produce dissolved organic matter or affect aggregation and export.

Size-based theory provides one bridge between identity and rates. Predator–prey size ratios are a major determinant of feeding efficiency and prey selection across planktonic consumers (Hansen *et al.,* 1994, 1997). However, size alone is insufficient. Feeding mode, encounter strategy, handling time, hydromechanical disturbance, metabolic cost and predation risk impose trait trade-offs that can separate organisms of similar size into very different functional roles (Kiørboe, 2011). A tintinnid ciliate filtering small motile prey, an aloricate ciliate feeding on nanoflagellates, and a pallium-feeding dinoflagellate attacking a diatom chain occupy different functional positions even when they are grouped into the same size class. Size spectra help models, but functional traits determine how carbon actually moves.

Grazer identity also changes the likely fate of carbon. Small ciliates feeding on picoplankton and nanoflagellates may retain carbon within rapidly recycling microbial pathways; larger ciliates and dinoflagellates can transfer carbon to copepods and other metazoans; pallium- or peduncle-feeding dinoflagellates may rupture or partially digest large prey, increasing dissolved and particulate by-products; and selective grazing on bloom-forming taxa can determine whether production is recycled locally, transferred upward or escapes toward aggregation and export. These alternative pathways are central to the ocean carbon cycle because consumer identity influences absorption, respiration, egestion, nutrient regeneration, trophic transfer and export efficiency (Steinberg and Landry, 2017). Thus, the question is not only how much phytoplankton mortality occurred, but which trophic route was opened by that mortality.

Heterotrophic dinoflagellates are a useful example because their ecological role is often underestimated by routine sampling. Many can ingest relatively large prey, including diatoms and other phytoplankton that are not efficiently consumed by smaller ciliates (Jeong, 1999; Sherr and Sherr, 2007). Some use specialized feeding mechanisms such as pallium feeding or peduncle feeding, which change the relationship between cell size and prey size. Their importance is therefore not captured simply by abundance. In a dilution experiment, ingestion by these forms cannot be measured from the dilution slope alone. It is inferred more defensibly when prey-specific loss of large target cells is coupled with direct observations of feeding structures, food vacuoles, prey remnants, labelled prey uptake or sorted-grazer prey signatures. A rare or moderately abundant dinoflagellate assemblage can impose strong mortality on large phytoplankton and alter bloom fate, but the mechanism must be supported by evidence beyond chlorophyll loss.

Ciliates raise a different problem. They are often abundant and important consumers of bacteria, pico- and nanoplankton (Sherr and Sherr, 1987, 2002), but their functional diversity matters. Aloricate oligotrichs and choreotrichs, tintinnids and mixotrophic ciliates differ in prey range, lorica protection, swimming behavior and preservation response. Tintinnids are attractive model organisms because the lorica provides a visible structure and many species can be identified with greater confidence than naked ciliates (Dolan *et al.,* 2013). Yet the same lorica can bias perception: structurally robust taxa may be overrepresented in preserved samples relative to fragile forms. Thus, even within ciliates, the observed community may not be the living functional community.

Mixoplankton are not merely a taxonomic complication; they are a conceptual challenge. Conceptual models of mixotrophy (Stoecker, 1998; Flynn and Mitra, 2009) and later functional classifications (Mitra *et al.,* 2016; Leles *et al.,* 2017, 2021; Mitra *et al.,* 2023) show that acquired phototrophy, constitutive mixotrophy and prey-dependent nutrition can produce different ecological strategies. Modelling and synthesis studies also indicate that mixotrophy can modify microbial trophic structure, trophic transfer efficiency and vertical carbon flux (Mitra *et al.,* 2014; Ward and Follows, 2016). Measuring grazing, primary production and dissolved nutrient uptake by mixoplankton cannot be achieved from a single dilution slope. A targeted design would need to combine dilution-derived prey loss with photosynthetic performance, light and nutrient treatments, prey-tracer uptake, pigment or plastid evidence, and, where possible, molecular or imaging separation of functional types. Different functional categories of mixoplankton should therefore be treated separately when they are abundant enough to influence the result; otherwise they should be reported explicitly as unresolved mixed trophic components.

The goal should not be a species-by-species rate matrix for the global ocean. That is unrealistic and not required for most ecological or modelling purposes. The useful middle ground is grazer-resolved functional inference. A minimal partition will vary by system, but defensible categories include small flagellate grazers, aloricate ciliates, tintinnids, heterotrophic dinoflagellates, mixoplankton functional types, nauplii or other metazoan micrograzers when present, and rhizarians or other large fragile protists where relevant. Within heterotrophic dinoflagellates, separating engulfment, pallium and peduncle feeders is useful when observations allow it. Rates should then be mapped onto functional groups or trait classes defined by prey-size range, feeding mode, trophic strategy, motility, mixotrophic capacity, growth efficiency and environmental sensitivity. This approach would allow dilution experiments, imaging, microscopy and molecular tools to contribute to a shared currency: not every species, but not a black box either.

## IMAGING AND METABARCODING: WHY MORE IDENTITY DOES NOT AUTOMATICALLY MEAN MORE FUNCTION

High-throughput imaging has changed the scale at which plankton can be observed. Instruments such as FlowCAM and Imaging FlowCytobot can generate large image archives, quantify size distributions, detect temporal changes and support semi-automated classification (Sieracki *et al.,* 1998; Olson and Sosik, 2007; Sosik and Olson, 2007; Álvarez *et al.,* 2014). These advances are particularly valuable for plankton groups that vary rapidly in time or are difficult to count manually at adequate frequency. They also make it possible to connect community structure to physical and chemical variability at resolutions that would be impossible with microscopy alone.

Imaging, however, does not solve the rate problem by itself. An image class is not a trophic flux. A time series of ciliate-like objects may reveal phenology, abundance and size, but it does not tell us whether those ciliates were feeding, what prey they consumed or how much carbon they processed. Images can infer traits such as size, shape and sometimes pigmentation or food vacuoles, but ingestion and growth remain processes. They require either direct observation, experimental calibration, prey tracking or modelling assumptions. Without independent rate or prey-linking evidence, automated imaging risks producing a very precise description of state variables without equivalent functional interpretation.

Automated classification also brings its own ecological biases. Machine-learning classifiers inherit the taxonomic conventions and sampling biases of their training sets. They often perform well on common, morphologically distinctive classes but poorly on rare, fragile, transitional or poorly labelled forms. Domain shift can occur when a classifier trained in one region, season or instrument setting is applied elsewhere. For microzooplankton, where diagnostic characters may be subtle and preservation can deform morphology, these biases are not trivial (Calbet, 2026). Automated identity must therefore be treated as probabilistic evidence, not as a final ecological truth. Benchmark image sets such as WHOI-Plankton have accelerated classifier development and intercomparison, but they also illustrate the dependence of automated identity on training labels, instrument settings and class definitions (Orenstein *et al.,* 2015).

Molecular approaches have produced an even deeper change by revealing diversity that microscopy underestimates or cannot reliably resolve. Tara Oceans and related surveys demonstrated extensive eukaryotic plankton diversity and strong biogeographic structure (de Vargas *et al.,* 2015). Regional metabarcoding has exposed rich protist assemblages in coastal waters and sediments (Massana *et al.,* 2015), and broader syntheses have emphasized the ecological reach of metabarcoding for protist diversity and the persistent technical and biological limitations of high-throughput metabarcoding (Santoferrara *et al.,* 2020; Burki *et al.,* 2021). These methods are essential for detecting cryptic taxa, life stages, parasites and rare lineages. Recent direct comparisons of metabarcoding and microscopy reinforce that the two approaches are complementary rather than interchangeable, especially when taxonomic resolution and abundance/biomass interpretation are required (Weydmann-Zwolicka *et al.,* 2024).

But sequence reads are not cells, biomass or rates. Amplicon abundance can be distorted by gene copy number, primer bias, extraction efficiency, cell size, life stage and reference-database limitations. Extracellular environmental DNA, including dissolved DNA and DNA associated with detritus, fecal material or damaged cells, may detect organisms that are not living, active components of the sampled assemblage. DNA or RNA collected from intact cells on a filter is different evidence and should not be conflated with dissolved environmental DNA. Metatranscriptomics and metagenomics add functional potential or activity proxies, but they still do not directly yield ingestion rates unless combined with experiments or prey-tracking approaches. Molecular data are therefore most powerful when they are linked to specific functional hypotheses: prey identity in sorted grazers, expression of feeding-related pathways, plastid retention, parasite interactions or cryptic taxa observed by other methods.

**Fig. 2:**
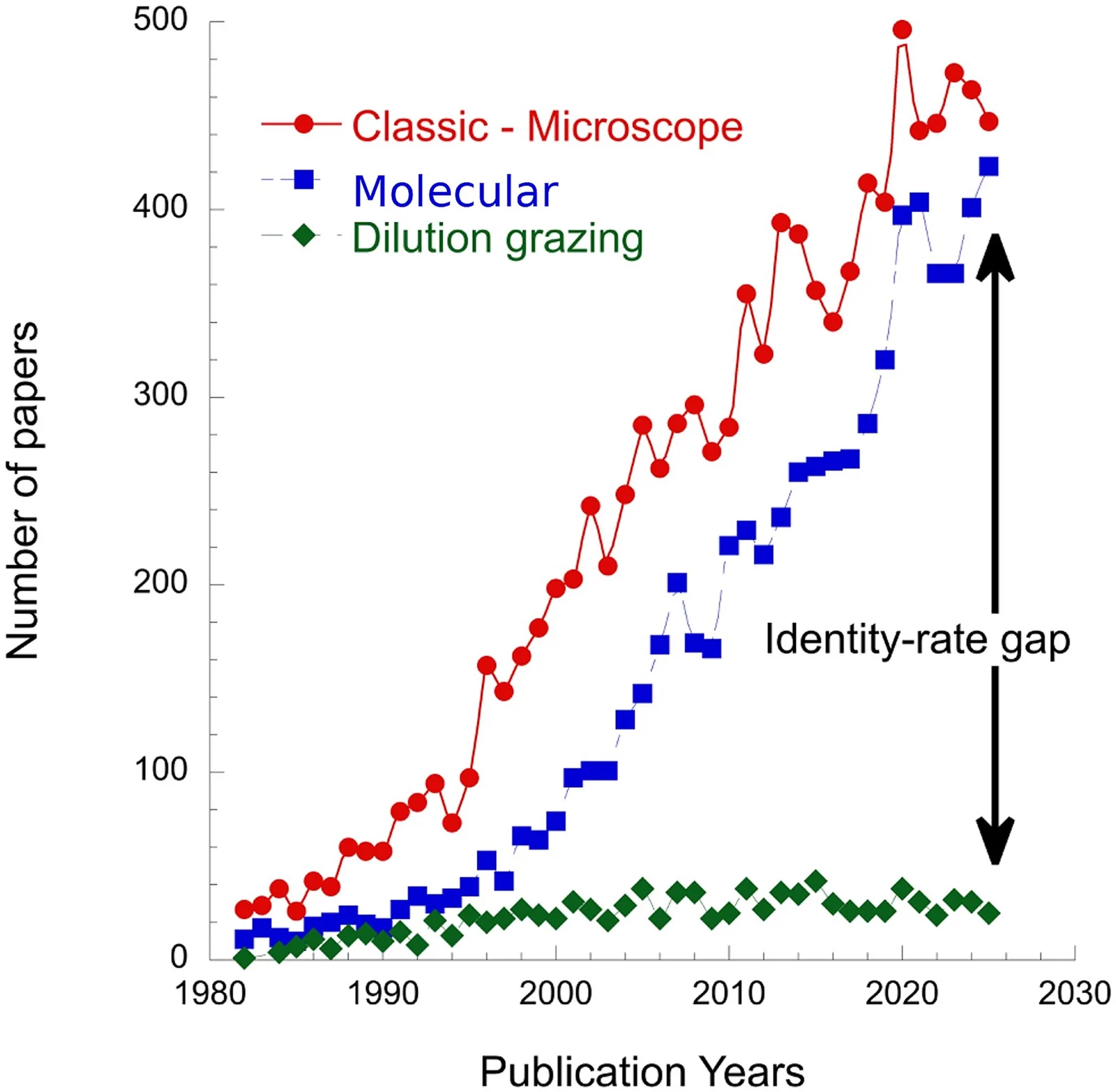
Illustrative literature trajectories behind the identity-rate gap. Annual publication counts are shown for three broad methodological streams: classical microscopic studies of protozoans or microzooplankton, molecular identity studies using planktonic protist terms together with molecular, DNA/RNA, sequencing, metabarcoding or omics terms and a conservative dilution-grazing stream. The categories are keyword-defined, partially overlapping and not normalized for database expansion; therefore, the plot should be read as a qualitative synthesis of literature trajectories rather than as a formal bibliometric meta-analysis or a census of all rate-relevant papers. Non-dilution rate approaches, including tracer, labelled-prey, food-vacuole and single-cell prey-content methods, are discussed in the text and Table I but are not plotted as a fourth curve because they are difficult to retrieve consistently with a single search string. The main message is the relative direction of change: molecular and identity-rich work is increasing rapidly, classical microscopy remains active, and the conservative dilution-grazing stream appears comparatively stable. For the descriptors used in the search see Supplementary Table 1. Alt-text: Line graph showing increasing annual counts for microscopy and molecular plankton-identity studies, lower and relatively stable counts for dilution-grazing studies, and an arrow marking the identity-rate gap between identity-rich and rate-oriented literature streams.

The literature trajectory mirrors this methodological divergence, but Fig. 2 should be read cautiously. It provides an illustrative comparison of annual publication counts for three broad methodological streams: dilution-based grazing estimates, microscopy-based studies of protozoans or microzooplankton, and molecular studies using terms such as metabarcoding, molecular, sequencing or omics in combination with planktonic protist terms. The pattern is not a formal bibliometric analysis and should not be interpreted as a count of all rate-relevant papers. Categories are search-term dependent, partially overlapping and affected by growth in database coverage. Because the dilution-grazing search is deliberately conservative, apparent stability in that stream should not be read as a decline of all rate-oriented microzooplankton research. Its qualitative meaning is narrower: identity-rich molecular work is expanding rapidly, classical microscopy remains highly active, and the conservative dilution-grazing stream appears comparatively stable. The identity-rate gap is therefore defined here by the weakness of direct linkage between identity and function, not by the absolute number of papers in any single method category.

**Fig. 3:**
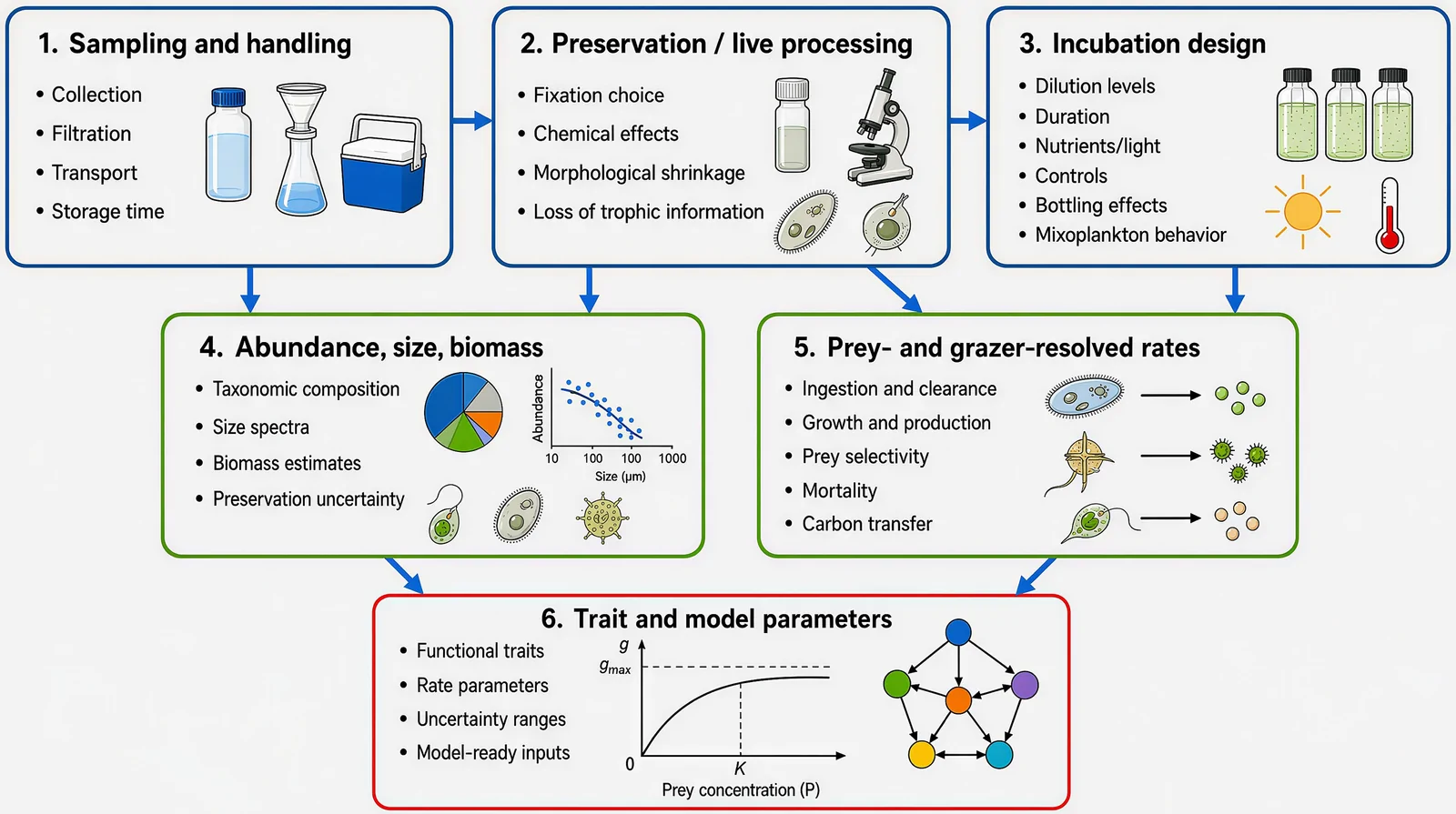
Methodological cascade from sampling to model inference. Preservation, handling and incubation artifacts can propagate through abundance, biomass and rate calculations into carbon-cycle parameters. The figure emphasizes that uncertainty should be reported and propagated rather than hidden inside state variables or bulk grazing coefficients. Alt-text: Flow diagram with six linked boxes showing sampling and handling, preservation or live processing, incubation design, abundance/size/biomass estimates, prey- and grazer-resolved rates, and trait or model parameters.

The practical implication is that samples should not be treated as either imaging samples, molecular samples or rate samples. Whenever the objective is functional interpretation, the relevant measurements must be paired within the same environmental and experimental context (Table II). Imaging can describe organisms and traits, molecular tools can reveal cryptic diversity or prey signatures, preserved microscopy can provide continuity with historical datasets, and incubations or tracer approaches can estimate rates. None of these layers can be assumed to provide a stable universal conversion for samples collected elsewhere, because grazer assemblages, prey fields, physiological state, preservation response, instrument settings and incubation artifacts vary among systems and seasons. The priority is therefore not to create general correction factors, but to design studies in which identity, biomass, prey composition, rates and uncertainty are measured together and interpreted within explicit limits (Fig. 3).

**Table II:** Minimum data package and functional partition for model-facing microzooplankton rate studies

Component	Minimum information	Why it matters
Experimental design	Dilution levels, bottle volume, duration, light, temperature, nutrient additions, filtration/pre-screening, controls and replicates (e.g. whether nutrients were added to all bottles).	Defines the perturbation and the assumptions behind inferred rates.
Prey state	Chlorophyll, flow-cytometric groups, size classes, pigments, image categories or molecular prey identity (e.g. Prochlorococcus, Synechococcus, picoeukaryotes, diatoms).	Identifies which prey pool the rate actually represents.
Grazer state	Microscopy, imaging, live observations and/or molecular identity of ciliates, heterotrophic dinoflagellates, mixoplankton, nauplii and other grazers; pre/post-incubation where possible.	Links bulk or prey-specific mortality to plausible consumers and identifies bottle-driven grazer changes.
Functional partition	Report the grazer categories that can be resolved in the study, such as small flagellate grazers, aloricate ciliates, tintinnids, heterotrophic dinoflagellates, mixoplankton functional types, nauplii/metazoan micrograzers and large fragile protists where relevant.	Prevents the trait framework from remaining abstract and makes clear which groups can be assigned separate rates, constraints or uncertainties.
Preservation metadata	Fixative, concentration, buffering, storage time, temperature, settling or filtration workflow.	Allows uncertainty in abundance, biomass and identity to be evaluated.
Rate resolution	Bulk community rate, prey-specific rate, grazer-specific ingestion, functional-group rate or model parameter (e.g. chlorophyll mortality, Synechococcus loss or ingestion of live fluorescently labelled algae, LFLA).	Prevents incompatible rates from being merged and defines the level of inference.
Complementary rate anchors	Report whether non-dilution methods were used, such as fluorescent-prey uptake or disappearance, food-vacuole/prey-content inspection, sorted-grazer prey DNA/RNA, stable-isotope or other tracers, flow-cytometric prey-loss measurements or behavioral imaging; include prey type, incubation time, concentration, sorting/counting basis and conversion to carbon where applicable.	Makes clear which claims are direct observations, prey-specific losses, grazer-linked ingestion proxies or model-facing constraints, and prevents complementary approaches from being merged as if they were the same rate currency.
Trait variables	Prey-size range, maximum ingestion, clearance rate, half-saturation constant, growth efficiency, feeding mode, mixotrophic strategy, temperature and light dependence; measured vs inferred/fitted.	Makes empirical results usable in trait-based and biogeochemical models.
Uncertainty	Replicate error, regression uncertainty, taxonomic uncertainty, preservation uncertainty, prey-conversion uncertainty and model-fitting uncertainty.	Allows rates to be propagated into synthesis and prediction and prevents apparent precision from hiding methodological uncertainty.

## PRESERVATION AS A HIDDEN UNCERTAINTY IN BOTH IDENTITY AND RATES

The preservation problem deserves a central place in the future agenda because it affects the state variables from which rates and models are built (Table I). Microzooplankton ecology still lacks a universally reliable fixative. Lugol’s iodine, formaldehyde, glutaraldehyde, protargol-based stains and combined protocols each preserve some features while damaging or obscuring others. This is not a technical footnote. It affects abundance, cell volume, biomass, food-vacuole visibility, chloroplast fluorescence, DNA compatibility and taxonomic recognition.

The quantitative evidence is substantial. Comparative and experimental studies have shown that fixation can cause strong shrinkage, swelling, loss of fragile taxa and altered detectability, while storage time and molecular compatibility vary among protocols (Bloem *et al.,* 1986; Choi and Stoecker, 1989; Vaulot *et al.,* 1989; Sherr and Sherr, 1993; Leakey *et al.,* 1994; Stoecker *et al.,* 1994; Pfister *et al.,* 1999; Chaput and Carrias, 2002; Karayanni *et al.,* 2004; Sano *et al.,* 2020). A recent synthesis concluded that fixation acts as a selective ecological filter rather than a neutral preservation step, with taxon-, fixative- and storage-dependent effects that can cascade into biomass and carbon-flux estimates (Calbet, 2026). The most important message for the present perspective is that preserved communities are not simply delayed observations of living communities; they are chemically and physically transformed assemblages.

This matters directly for the identity-rate gap. If fragile grazers are lost, shrunken or misclassified, the apparent community used to interpret a grazing rate is biased before rate inference begins. If aloricate ciliates shrink more than loricate forms, biomass spectra shift. If chloroplast fluorescence is lost, mixotrophs may be misclassified. If food vacuoles collapse or prey visibility changes, direct evidence of trophic links disappears. If storage time differs among samples, spatial or seasonal differences may partly reflect laboratory history. These effects do not merely add noise; they can shift the inferred relative importance of functional groups.

The biomass problem is especially acute because many ecological calculations convert volume to carbon. Cell volume is among the properties most affected by preservation, and carbon-volume relationships are nonlinear (Montagnes *et al.,* 1994; Menden-Deuer and Lessard, 2000; Menden-Deuer *et al.,* 2001; Modigh and Castaldo, 2005). A moderate shrinkage in volume can therefore produce a large change in biomass, and biomass-normalized rates can be distorted in both numerator and denominator. Imaging-based comparisons further show that preservation can alter particle counts and size distributions, not only taxonomic labels (Zarauz and Irigoien, 2008; Jakobsen and Carstensen, 2011). When such estimates enter global syntheses or model parameterizations, preservation uncertainty becomes a hidden component of carbon-cycle uncertainty.

The solution is not to search for a perfect fixative. The literature does not support one. Instead, the field should adopt preservation-aware ecology. Studies should report fixative type, concentration, buffering, storage time, temperature, settling or filtration procedure and whether live comparisons were made. Where possible, live observations should be paired with fixed counts, and taxon-specific uncertainty ranges should be propagated into biomass and rate estimates. For model-facing datasets, preservation metadata should be treated as part of the data, not as supplementary trivia.

This argument should not duplicate specialized reviews of preservation artifacts. Its role here is narrower: to show that identity and rates are connected by fragile state variables. A grazing rate cannot be fully interpreted if the grazer assemblage has been selectively transformed before observation. A molecular profile cannot be equated with a living food web if biomass and activity are not measured. Preservation is therefore one of the hidden bridges between methodological practice and ecological inference.

## GEOGRAPHY, ENVIRONMENTAL CONTEXT AND THE DANGER OF GLOBAL EXTRAPOLATION

The global importance of microzooplankton grazing is well established, but global parameterization remains weak. Functional datasets are easiest to obtain at coastal stations, time-series sites and regions accessible to shipboard experimentation and trained taxonomists. Remote oligotrophic gyres, polar waters, oxygen minimum zones, deep chlorophyll maxima and transient frontal systems are less well represented. This geographical imbalance matters because microzooplankton function is not expected to scale uniformly across ocean regimes. Recent regional and event-focused rate studies illustrate the value of retaining high-resolution grazing measurements under contrasting coastal and heatwave conditions, while also confirming that local environmental context shapes interpretation (Wen *et al.,* 2023; Landry *et al.,* 2024b).

Productive coastal systems, oligotrophic gyres and polar waters impose different energetic constraints. In coastal blooms, prey can be abundant and large, grazer responses may lag, and heterotrophic dinoflagellates or mesozooplankton may dominate particular loss pathways, creating potential loopholes in microzooplankton grazing control (Irigoien *et al.,* 2005). In oligotrophic systems, prey concentrations are low, picocyanobacteria and small eukaryotes often dominate, mixotrophy may be advantageous, and grazing estimates depend strongly on whether the measured prey pool is bulk chlorophyll, flow-cytometric picoplankton or specific prey groups (Zubkov and Tarran, 2008; Calbet *et al.,* 2012; Hartmann *et al.,* 2012). In polar systems, seasonality, low temperature, extreme light cycles, ice dynamics and bloom phenology create different coupling between primary production and protistan herbivory; dilution experiments and polar mixotrophy syntheses show that grazing can be strong in some contexts but weak, nonlinear or difficult to interpret during blooms dominated by particular taxa or during strong seasonal transitions (Calbet *et al.,* 2011; Sherr *et al.,* 2013; Stoecker and Lavrentyev, 2018). A mean global grazing fraction is therefore useful as a synthesis statistic but insufficient as a predictive framework.

Environmental context also modifies traits. Temperature changes growth, respiration and ingestion, but not necessarily with identical sensitivities across trophic modes. Nutrient limitation alters prey quality and can shift mixotrophs toward phagotrophy for nutrient acquisition. Light influences the balance between phototrophy and feeding in mixoplankton. Turbulence can affect encounter rates, prey handling and predator–prey behavior. These drivers interact rather than act independently, a point emphasized by climate-focused syntheses of microzooplankton grazing and community structure (Caron and Hutchins, 2013). A trait-rate value measured in one context may therefore not transfer to another without information on prey field, grazer state and environment.

The model connection clarifies why this matters. Size-structured and trait-based models have improved the representation of plankton diversity and emergent biogeography (Follows *et al.,* 2007; Ward *et al.,* 2012, 2014). However, grazer parameters remain weakly constrained relative to their influence on biomass, trophic transfer and export. Zooplankton syntheses show that carbon processing by protistan and metazoan consumers involves ingestion, assimilation, respiration, excretion, egestion and growth, with regional variability in export pathways (Steinberg and Landry, 2017). Rohr *et al.* (2023) showed that phytoplankton-specific loss rates to zooplankton grazing are the largest source of inter-model uncertainty in CMIP6 marine carbon-cycle representation. Meyjes *et al.* (2024) showed that spatial variability in zooplankton grazing parameters can change simulated carbon export. These studies underscore that grazing is not a minor tuning term. It is a control point in modelled carbon flow.

For microzooplankton ecology, the challenge is to provide models with variables that are both biologically meaningful and usable. Most marine ecosystem and Earth system models will not include separate parameters for every ciliate, dinoflagellate, mixoplankton or prey lineage. They usually operate with a small number of plankton functional types, size classes or prey-size-dependent mortality functions. The useful contribution is therefore not maximal taxonomic detail, but rate information that can constrain those simplified structures: prey-size-specific mortality, functional response parameters, temperature sensitivities, growth efficiencies, trophic-transfer efficiencies and uncertainty ranges. Models also need to know whether a rate represents total chlorophyll loss, prey-specific loss, targeted grazer-specific ingestion or inferred functional-group activity. Without that distinction, empirical rates are difficult to compare and easy to misuse.

The bridge to models should therefore separate four classes of information. First are direct empirical rates, such as prey-specific mortality or ingestion under stated experimental conditions. Second are convertible rates, such as chlorophyll-based losses that require assumptions about prey carbon, grazer identity and growth efficiency. Third are trait constraints, such as prey-size range, maximum ingestion, clearance rate or temperature sensitivity, which can bound parameter values even when they are not inserted directly into models. Fourth are validation variables, such as community composition, biomass spectra or bloom timing, which test whether model behavior is plausible. Mixing these categories obscures uncertainty because users cannot tell whether variation reflects biology, preservation, incubation design, conversion assumptions or model fitting; distinguishing them makes microzooplankton data more usable.

A future global synthesis should therefore be more than a map of mean grazing rates. It should ask which functional groups dominate grazing under which environmental regimes, how preservation and incubation biases vary among regions, how mixoplankton change the interpretation of phytoplankton mortality, and which rate variables most reduce model uncertainty. The realistic near-term target is not uniform global coverage of every interaction, but a strategically distributed set of well-documented studies across major biomes, seasons and recurring observing sites. This is a different kind of global synthesis: not only more points on a map, but better connections between biology, measurement and prediction.

## A PRACTICAL AGENDA FOR THE NEXT DECADES

Closing the identity-rate gap requires a practical, tiered measurement architecture rather than a single new method. Classical dilution experiments should remain central because they measure emergent trophic effects in natural communities. But they should increasingly be embedded within designs that identify the organisms and traits responsible for those effects when the research question demands that level of inference. The aim is not methodological replacement and not universal maximal complexity at every station. Broad surveys can remain Tier 1; more elaborate Tier 2 or Tier 3 designs should be reserved for questions that require grazer attribution, targeted ingestion or model-facing trait constraints. It is coordinated resolution: matching the number of measurements to the rate question being asked (Fig. 1).

First, dilution experiments should become identity-aware incubations when they are used to support identity-aware claims. At minimum, studies should report prey composition, grazer composition, nutrient status, incubation light and temperature, bottle volume, dilution levels and post-incubation community changes. Where feasible, rates should be resolved by prey size class, flow-cytometric group, pigment group, image class or molecularly identifiable prey. Several studies have already implemented parts of this agenda, including taxon- or population-focused dilution designs and sequence-based modifications (Worden and Binder, 2003; Landry *et al.,* 2024a; Gu *et al.,* 2026). Prey-tracer approaches, including LFLA, can help identify active grazers and prey-specific ingestion when their assumptions and prey-selectivity limitations are tested explicitly (Martínez *et al.,* 2014). Bulk chlorophyll grazing rates remain useful; the point is not that they are routinely misrepresented, but that their inferential level should remain visible when they are compared with prey-resolved or grazer-linked measurements.

Second, non-dilution and single-cell rate approaches should be used explicitly as complementary rate anchors rather than as decorative identity layers. Disappearance of fluorescently labelled bacteria or algae, prey uptake assays, food-vacuole and prey-content observations, sorted-grazer prey DNA/RNA, stable-isotope and other tracers, flow-cytometric prey-disappearance and behavioral imaging each constrain a different part of the identity-rate problem (Sherr and Sherr, 1987; Martínez *et al.,* 2014; Beatty *et al.,* 2025). These methods often have stronger grazer- or prey-level specificity than bulk chlorophyll slopes, but narrower prey realism, calibration or denominator limits. The practical standard is to state which rate currency each method supports and how it is linked to the dilution experiment or environmental sample.

Third, identity and rate measurements should be paired at the scale at which inference is made. A grazing estimate obtained from one bottle cannot be fully interpreted from a taxonomic description obtained under different conditions, and an image class or sequence variant observed in one system cannot be assigned a fixed trophic role in another. The same organism may differ in feeding activity, prey preference, growth efficiency or preservation response depending on food availability, nutrient status, light, temperature, community composition and handling. This limited transferability does not make the information useless for models. It means that results should be reused as conditional parameter constraints, uncertainty ranges and tests of model structure, not as universal constants attached to a taxon or method.

Fourth, preservation uncertainty should be reported and propagated (Calbet, 2026). A dataset that includes rates but omits fixative details, storage time and counting workflow is incomplete. Journals and data repositories should require preservation metadata for microzooplankton studies in the same way that they require temperature, location or sampling date. This does not solve preservation bias, but it makes the uncertainty visible and reusable.

Fifth, microzooplankton trait-rate repositories should be designed for models from the start, but they should preserve the distinction between observations, fitted parameters and inferred constraints. Variables should include ingestion rate, clearance rate, prey-size range, half-saturation constant, maximum growth rate, growth efficiency, feeding mode, mixotrophic strategy, temperature response, light dependence, prey quality response and uncertainty. Each value should be linked to taxonomic identity, experimental design and environmental context. The repository should distinguish observed rates from inferred or model-fitted parameters and should indicate the level of evidence supporting each grazer-prey link.

Sixth, training and culturing must remain biological. Automated classification and bioinformatics can expand throughput, but they cannot replace organismal judgment. Cultures are essential for measuring functional traits, validating prey tracers, testing molecular or imaging proxies and identifying physiological mechanisms. At the same time, many microzooplankton cannot be maintained in stable culture, and cultured strains may change feeding behavior, prey preference or thermal response under laboratory conditions. Culture-derived traits should therefore be treated as mechanistic anchors that need field validation, not as complete substitutes for natural assemblage measurements. The future microzooplankton ecologist should be able to recognize living protists, understand fixation artifacts, design incubations, evaluate molecular data and communicate with modelers. Classical taxonomy, live observation and culturing are not nostalgic practices. They are the biological ground truth that prevents high-throughput data from becoming detached from organisms.

The agenda can be summarized simply: preserve the strengths of the bottle, but stop treating the bottle as the whole experiment or as the only rate framework available. A next-generation study should ask not only what the grazing rate was, but what level of inference the design actually supports: bulk prey mortality, prey-group mortality, direct prey uptake or disappearance, plausible grazer attribution, targeted grazer-prey ingestion, or model-facing parameter constraints. That clarification makes complex experiments useful without pretending that every bottle or every single-cell proxy solves the entire food web.

## CONCLUSIONS: BEYOND THE BOTTLE DOES NOT MEAN WITHOUT THE BOTTLE

Microzooplankton ecology does not need another general argument that protistan grazers matter. That case has been made. The field now needs a more demanding form of evidence: rates connected to identities, identities connected to traits, traits connected to environmental context, and all of these connected to models with explicit uncertainty.

Dilution experiments should remain part of the field because they measure emergent trophic effects that imaging and sequencing cannot yet replace. But their interpretation should be more explicit, more diagnostic and more frequently paired with independent evidence. Similarly, imaging and molecular methods should not be treated as replacements for rates. They are powerful identity tools that become functionally transformative only when anchored to measurements of feeding, growth, mortality or prey tracking.

The next decades should therefore be defined by coordinated resolution rather than by a choice between bottles and high-throughput identity tools. The aim is not to abandon the bottle, but to open it: to connect the rates it revealed with the organisms, traits, behaviors and environments that produce them, while acknowledging which interactions remain inferred. Microzooplankton ecology will be most useful to ocean science when it can say not only how much carbon was consumed, but by whom, under what conditions, with what level of uncertainty and with what consequences for the food web and the carbon cycle.

## Supplementary Material

Supplementary_Table_1_FINAL_revised_fbag070

## Data Availability

No new measurements are reported in this paper.
