# Supplementary material for: Beyond the bottle: exposing the identity-rate gap in microzooplankton ecology
Source: J Plankton Res. 2026 Sep 1;48(5):fbag070. doi: 10.1093/plankt/fbag070 (PMC13533193; doi:10.1093/plankt/fbag070)
Supplement: Supplementary file 1 [file supplementary_table_1_final_revised_fbag070.docx]

**Supplementary Table 1.**

Search descriptors used for the illustrative literature trajectories in Figure 2.

The searches were conducted in Scopus and were intended to generate qualitative, keyword-defined trajectories, not a formal bibliometric dataset. Categories are partially overlapping and sensitive to database coverage, terminology and search-string design. The dilution-grazing trajectory is deliberately treated as a conservative rate-oriented stream and should not be interpreted as the complete literature on microzooplankton functional rates. Non-dilution and single-cell rate approaches were not plotted as a fourth curve because terms for labelled-prey uptake, food vacuoles, prey-content analysis, tracer incubations, flow-cytometric prey loss and behavioural imaging are too heterogeneous to retrieve consistently with a single search string.

For dilution-grazing studies

TITLE-ABS-KEY ((dilution W/3 (graz* OR microzooplankton OR micro-zooplankton OR phytoplankton OR mortality OR growth)) OR "Landry-Hassett" OR "Landry and Hassett" OR "dilution technique" OR "dilution grazing" OR doi:10.1007/BF00397668) AND TITLE-ABS-KEY (marine OR ocean* OR seawater OR coastal OR estuar* OR pelagic OR plankton*)

For molecular studies of field plankton identification

TITLE-ABS-KEY ((protozoa* OR protist* OR "microbial eukaryote*" OR "planktonic eukaryote*" OR microzooplankton OR micro-zooplankton OR ciliat* OR tintinnid* OR dinoflagellat* OR "heterotrophic flagellate*" OR nanoflagellat* OR radiolarian* OR foraminifer* OR "marine alveolate*" OR MALV OR mixoplankton OR mixotroph*) AND (molecular OR DNA OR RNA OR rDNA OR rRNA OR "18S" OR "SSU rRNA" OR "small subunit" OR metabarcod* OR barcod* OR sequencing OR "high-throughput sequencing" OR "next-generation sequencing" OR NGS OR Illumina OR MiSeq OR pyrosequenc* OR "clone librar*" OR DGGE OR "T-RFLP" OR qPCR OR FISH OR "CARD-FISH" OR "oligonucleotide probe*" OR eDNA OR "environmental DNA" OR metagenom* OR metatranscriptom*) AND (plankton* OR marine OR ocean* OR seawater OR coastal OR estuar* OR pelagic OR mesocosm* OR "field sample*" OR "environmental sample*"))

For microscopy/classic studies of field plankton identification

TITLE-ABS-KEY ((microzooplankton OR micro-zooplankton OR protozoa* OR protist* OR ciliat* OR tintinnid* OR oligotrich* OR choreotrich* OR dinoflagellat* OR "heterotrophic flagellate*" OR nanoflagellat* OR "microbial eukaryote*" OR "planktonic eukaryote*" OR mixoplankton OR mixotroph*) AND (marine OR ocean* OR seawater OR coastal OR estuar* OR pelagic OR plankton* OR "water column" OR "field sample*" OR "natural assemblage*") AND (microscop* OR "light microscop*" OR "inverted microscop*" OR "epifluorescence microscop*" OR "phase contrast" OR Utermohl OR Utermöhl OR "settling chamber*" OR "sedimentation chamber*" OR Lugol* OR formalin OR formaldehyde OR formol OR glutaraldehyde OR "fixed sample*" OR preserv* OR fixative* OR "cell count*" OR enumerat* OR abundance OR biomass OR biovolume OR protargol OR "quantitative protargol stain*" OR QPS OR "silver impregnation" OR DAPI OR "acridine orange"))
